# Zika Virus Infection and Prolonged Viremia in Whole-Blood Specimens

**DOI:** 10.3201/eid2305.161631

**Published:** 2017-05

**Authors:** Jean Michel Mansuy, Catherine Mengelle, Christophe Pasquier, Sabine Chapuy-Regaud, Pierre Delobel, Guillaume Martin-Blondel, Jacques Izopet

**Affiliations:** Centre Hospitalier Universitaire de Purpan, Toulouse, France (J.M. Mansuy, C. Mengelle, C. Pasquier, S. Chapuy-Regaud, P. Delobel, G. Martin-Blondel, J. Izopet);; Institut National de la Santé et de la Recherche Médicale, Toulouse (C. Pasquier, S. Chapuy-Regaud, P. Delobel, G. Martin-Blondel, J. Izopet);; Université Paul Sabatier, Toulouse (C. Pasquier, S. Chapuy-Regaud, P. Delobel, J. Izopet)

**Keywords:** Zika virus, viruses, whole blood, plasma, viremia, RNA, blood donation, vector-borne infections

## Abstract

We tested whole-blood and plasma samples from immunocompetent patients who had had benign Zika virus infections and found that Zika virus RNA persisted in whole blood substantially longer than in plasma. This finding may have implications for diagnosis of acute symptomatic and asymptomatic infections and for testing of blood donations.

Since cases of severe neurologic disorders among adults (*1*) and fetal abnormalities (*2*) linked to Zika virus infections were initially reported, the World Health Organization has deemed the Zika virus outbreak a “public health emergency of international concern” and has raised Zika virus to the same level of concern as Ebola virus. In response, medical authorities from many countries have released advice and guidelines regarding prevention and diagnosis to contain the spread of this virus and guidelines regarding safety of whole blood and blood components. In August 2016, the Food and Drug Administration announced universal testing for Zika virus RNA in donated whole blood and blood components taken in the United States and its territories using a qualitative molecular assay on plasma specimens (*3*).

In Europe, advice on Zika virus regarding the safety of substances of human origin (*4*) has been applied in France since February 15, 2016. A qualitative individual molecular test for Zika virus RNA in plasma specimens is being used on whole-blood specimens from blood donors living in Guadeloupe and Martinique, 2 overseas administrative areas where Zika virus is autochthonous. Furthermore, in mainland France and in French overseas areas where no active Zika virus transmission exists, and since the beginning of the Zika virus outbreak in 2015, blood donors who have recently visited areas or countries with ongoing Zika virus transmission are subject to a 28-day temporary deferral after their departure from these areas, a period twice the assumed maximum incubation period for Zika virus. Similarly, temporary deferral applies to blood donors who have a sex partner who has been recently infected or potentially exposed to a confirmed or suspected Zika virus infection within the previous 3 months.

We report results from a 2016 longitudinal follow-up of Zika virus RNA quantification in EDTA whole-blood and plasma samples taken from 5 immunocompetent patients (2 men, 33 and 70 years of age, and 3 women, 55, 58, and 67 years of age) and results from a point-to-point comparison of Zika viral loads on both EDTA whole-blood and corresponding plasma samples (27 pairs). We extracted RNA by using the MagNA Pure 96 instrument with the DNA and Viral NA Small Volume Kit (Roche Diagnostics, Meylan, France) (input and output volumes 200 and 100 µL). We quantified RNA by using the RealStar Zika RNA RT-PCR kit 1.0 (Altona Diagnostics GmbH, Hamburg, Germany) (limit of selection 2.48 log copies/mL). We always successfully detected the manufacturer’s internal control. All samples were collected from patients who had returned from the Caribbean or South and Central America and had had a benign form of Zika virus infection.

Results from the follow-up (18 whole-blood and 21 plasma samples) showed that the median duration of Zika virus was 22 (range 14–100) days in whole blood and 10 (range 7–37) days in plasma (p = 0.058). Mean viral loads of positive samples were 3.39 log copies/mL in whole blood (n = 13) and 2.52 log copies/mL in plasma (n = 6; p = 0.001). Viral loads in the last positive samples varied from 2.7 to 3.9 log copies/mL in whole blood and 2.2 to 2.8 log copies/mL in plasma (p = 0.06). Whole-blood samples from 2 patients remained positive at 14 and 63 days after their plasma samples had become negative (Figure, panel A).

**Figure:**
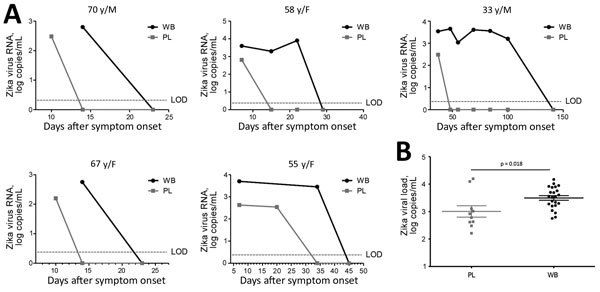
A) Zika virus viremia in whole blood and plasma from 5 immunocompetent patients in France (identified by sex and age, y) who had traveled to Central or South America or the Caribbean. B) Zika viral load in whole-blood (n = 23) and plasma (n = 10) samples from a point-to-point comparison of positive samples. Horizontal lines indicate mean +SE. LOD, limit of detection; PL, plasma; WB, whole blood.

The point-to-point comparison (18 pairs from the follow-up and 9 additional pairs) showed that Zika virus RNA was quantifiable in 23 whole-blood specimens but in only 10 plasma samples. Mean viral load was 3.50 (range 2.75–4.17) log copies/mL in whole blood and 3.01 (range 2.21–4.10) log copies/mL in plasma (p< = 0.018) (Figure, panel B).

These data show that Zika virus RNA persisted in whole blood after it disappeared in plasma. Similar results have been reported previously for West Nile virus, also a member of the *Flaviviridae* family (*5*,*6*), and for Zika virus with a qualitative in-house PCR (*7*).

Our data have 3 main consequences. First, for acute symptomatic infection, the use of whole blood extends the period of diagnosis. Second, for asymptomatic infections with a high likelihood of low viral load, virus detection in plasma might be less sensitive than detection in whole-blood specimens. Third, according to our data that show that viremia can persist for >28 days after symptom onset, recommendations used to reduce the risk for Zika virus transmission through blood or blood components should be modified. Potential options such as extending the deferral period or testing blood donations for Zika virus RNA in whole blood should be considered.

Overall, our data show that use of whole-blood specimens is much more sensitive than use of plasma samples to detect Zika virus RNA. These data could be useful in recommending the use of whole blood instead of plasma for the molecular diagnosis of acute symptomatic and asymptomatic Zika virus infections and for the safety of whole blood and blood components from donors, as well as for the safety of organs, tissues, and cells from deceased and living donors.
